# Toll-Like Receptor 3 in Solid Cancer and Therapy Resistance

**DOI:** 10.3390/cancers12113227

**Published:** 2020-11-02

**Authors:** Ximena Maria Muresan, Jan Bouchal, Zoran Culig, Karel Souček

**Affiliations:** 1 Department of Cytokinetics, Institute of Biophysics of the Czech Academy of Sciences, 612 65 Brno, Czech Republic; muresan@ibp.cz; 2 International Clinical Research Center, St. Anne’s University Hospital in Brno, 656 91 Brno, Czech Republic; zoran.culig@fnusa.cz; 3 Department of Clinical and Molecular Pathology, Institute of Molecular and Translational Medicine, Faculty of Medicine and Dentistry, Palacky University and University Hospital, 779 00 Olomouc, Czech Republic; jan.bouchal@upol.cz; 4 Department of Urology, Experimental Urology, Innsbruck Medical University, A-6020 Innsbruck, Austria; 5 Department of Experimental Biology, Faculty of Science, Masaryk University, 625 00 Brno, Czech Republic

**Keywords:** toll-like receptor 3, therapy resistance, cytokines, dsRNA, metastasis

## Abstract

**Simple Summary:**

Toll-like receptor 3 (TLR3) is a member of the TLR family, which has been extensively studied for the antiviral function and, therefore, its role in the innate and adaptive immune responses. It is highly expressed in the endosomes of antigen-presenting immune cells and epithelial cells. Several studies have demonstrated TLR3 expression in multiple neoplasia types including breast, prostate, and ovarian cancer. In this perspective, we focus on the mechanisms through which TLR3 can either lead to tumor regression or promote carcinogenesis as well as on the potential of TLR-based therapies in resistant cancer.

**Abstract:**

Toll-like receptor 3 (TLR3) is a member of the TLR family, which has been extensively studied for its antiviral function. It is highly expressed in the endosomes of antigen-presenting immune cells and epithelial cells. TLR3 binds specifically double-strand RNAs (dsRNAs), leading to the activation of mainly two downstream pathways: the phosphorylation of IRF3, with subsequent production of type I interferon, and the activation of NF-κB, which drives the production of inflammatory cytokines and chemokines. Several studies have demonstrated TLR3 expression in multiple neoplasia types including breast, prostate, and lung cancer. Most studies were focused on the beneficial role of TLR3 activation in tumor cells, which leads to the production of cytotoxic cytokines and interferons and promotes caspase-dependent apoptosis. Indeed, ligands of this receptor were proposed for the treatment of cancer, also in combination with conventional chemotherapy. In contrast to these findings, recent evidence showed a link between TLR3 and tumor progression, metastasis, and therapy resistance. In the present review, we summarize the current knowledge of the mechanisms through which TLR3 can either lead to tumor regression or promote carcinogenesis as well as the potential of TLR-based therapies in resistant cancer.

## 1. Introduction

### 1.1. Structure, Activation Pathway, and Cell Distribution of TLR3

Toll-like receptor 3 (TLR3) is a type of pathogen recognition receptor, which preferentially binds to double-strand RNA (dsRNA) derived from the viral genome, released from viral particles or damaged host cells [1].

The molecular structure of human TLR3 ectodomain (ECD) is characterized by a solenoid with 23 leucine-rich repeats assembled into a horseshoe shape, capped at each end by specialized structures known as Leucine-Rich Repeat N-terminal (LRR-NT) and C-terminal (LRR-CT) domains, which are thought to be critical for dsRNA recognition [2]. Although TLR3-ECD appears as monomeric in solution, its dimerization, controlled by LRR-CT, is necessary for the binding of dsRNA, thus underlining the importance of a dsRNA length of a minimum of 40 bp to be properly recognized [3]. Furthermore, the binding of dsRNA takes place only at acidic pH (pH equal to or lower than 6.5) [4] in line with the endosomal localization and activation of the receptor.

Once dsRNA is internalized into the cells and detected by TLR3 in the endosomal compartment, the signaling pathway of this receptor is activated. Among all TLR family members, TLR3 is the only receptor that does not use myeloid differentiation factor 88 (MyD88) as a signaling adapter [5]. Indeed, TLR3 transmits signals via the adaptor protein Toll-IL-1 receptor (TIR) domain-containing adaptor molecule-1 (TICAM-1) (also known as TIR domain-containing adapter inducing IFN-β (TRIF)). This leads to the activation of transcription factors, such as interferon regulatory factor 3 (IRF-3), NF-κB, or AP-1, leading finally to the induction of type I interferon (especially IFN-β), as well as to the production of inflammatory cytokines and chemokines [6,7]. The association of TLR3 and TRIF takes place after the phosphorylation of two tyrosine residues (Tyr759 and Tyr 858) on the cytoplasmic domain of the receptor [8]. The choice of the downstream signaling pathway to be activated is, therefore, given by the cytoplasmic domain of TLR3 and by TRIF. Indeed, TRIF can associate with tumor necrosis factor receptor (TNFR)-associated factor 3 (TRAF3) and promote the phosphorylation and activation of IRF3, followed by the production of IFNs [9]. In parallel, TRIF is also able to associate with TNFR-associated factor 6 (TRAF6) and receptor-interacting protein 1 (RIP1), inducing the activation of NF-κB and the production of inflammatory cytokines. In this context, all TLR signaling pathways lead to the activation of NF-κB. Consistent with this, a synthetic dsRNA analog Poly(I:C) stimulates IRF3 phosphorylation, but not NF-κB activation in TRAF6-/- MEFs [10].

Previous studies have reported TLR3 expression in various tissues and cell types. For instance, TLR3 was found to be expressed in immune cells, such as myeloid antigen-presenting DCs, macrophages, or NK cells [11,12]. On the other hand, TLR3 was also shown to be constitutively expressed in the human central nervous system, mainly in neurons, astrocytes, and microglia [13]. In neurons, TLR3 inhibits axonal growth [14]. TLR3 is also expressed in fibroblasts and several epithelial cells, such as the airway, corneal, cervical, and intestinal cells [5].

At a cellular level, TLR3 is expressed exclusively intracellularly in myeloid dendritic cells (DCs) [15], while in fibroblasts, macrophages, and some epithelial cell lines it is localized both on the cell surface and intracellularly in early endosomes [16]. The localization of TLR3 is dependent on an accessory endoplasmic reticulum (ER) resident protein named UNC93B1, which interacts with the transmembrane segment of the receptor and is responsible for its trafficking inside the cell [17]. Several studies have reported the importance of the transmembrane or cytoplasmic domain, as well as of the ectodomain (ECD) in governing the plasma membrane expression of the receptor, but regardless of its cell surface expression, it has been described that TLR3 activation requires the endosomal localization [18].

### 1.2. The Function of TLR3 in the Immune System and Epithelial Cells

The main function of TLR3 in immune cells is the induction of cross-presentation of antigens to CD8^+^ T cells. Striking evidence was offered by Schulz and colleagues [19]. The authors demonstrated that phagocytosis of viral material by CD8α^+^ dendritic cells, the major antigen-presenting cell subtype in priming antiviral cytotoxic T cells (CTL), leads to a high increase in CTL response (cross-priming) in mice, and this phenomenon is dependent on TLR3 activation. Given the expression of TLR3 in key sentinel cells of the innate immune system and its ability in recognizing viral RNA, multiple studies have been extensively focused on the contribution of this receptor to immunity. In vivo experiments on mice lacking TLR3 have shown the importance of this receptor in susceptibility to mouse cytomegalovirus (MCMV) infection and IFN production [20]. In parallel, it has been reported that TLR3 knockout in mice leads to uncontrolled replication and spread of herpes simplex virus (HSV) 2 to the central nervous system (CNV) [21]. Additionally, in humans, TLR3 appears to be crucial in protecting against HSV-1. Indeed, children born with TLR3 deficiency present a higher incidence of herpes simplex-associated encephalitis [22]. Moreover, there is other evidence which shows the importance of TLR3 in immune recognition of other viruses, such as poliovirus [23], encephalomyocarditis virus [24], or hepatitis C virus [25].

Interestingly, studies have shown that TLR3 might also be implicated in conditions such as type 1 diabetes [26], underlining the ability of this receptor in recognizing not only viral dsRNA but also endogenous dsRNA derived from necrotic cells. Furthermore, another important function of TLR3 signaling is its association with tumor cell growth. It is known, for instance, that TLR3 activation in host immune cells present in the tumor microenvironment induces the activation of cells, such as tumor-suppressive M1 macrophages or tumor-associated CD11b^+^Ly6G^+^ neutrophils, with final tumoricidal outcome [27,28].

## 2. Involvement of TLR3 in Cancer

### 2.1. Expression in Human Cancers and Its Relation to Clinical Outcome

In recent years, multiple studies have demonstrated that TLR3 expression and activation play a role in tumor progression. TLR3 expression-associated prognosis for each cancer type, together with other details, is depicted in Table 1. TLR3 overexpression is often associated with a good prognosis or good response to TLR3 agonists in patients affected by hepatocellular carcinoma, melanoma, lung adenocarcinoma, and renal cancer. In parallel, in other cancer types, such as prostate, oral, esophageal, or gastric cancer, TLR3 expression appears to be associated with poor prognosis.

These discrepancies and differences in clinical outcome could be given by the different nature of each tumor type and environment, as well as by the well-known heterogeneity between patients. Of note, the authors used different antibodies, mostly without stringent validation for immunohistochemistry on formalin-fixed paraffin-embedded tissues [29]. There is no clear correlation between TLR3-dependent prognosis and cancer type or intracellular localization of the receptor. It is known that this receptor has a cytoplasmic expression in endosomes or on the plasma membrane. However, critical observation of previously published IHC results show nuclear positivity of TLR3 in a certain percentage of tumor cells (see Table 1), but no precise interpretation of such pattern is available up-to-date. We hypothesize that the nuclear TLR3 signal could be a consequence of non-specific staining, although this aspect needs to be further investigated. This area will need further elaboration with proper antibodies as well as with other reliable methods. The study by Bonnin et al. may serve as a good example where convincing results were achieved by expression analysis of patient samples (immunohistochemistry and RT-PCR) and functional experiments in vitro and in vivo (several cell lines and TLR3 knock-out animals, respectively) [30].

### 2.2. TLR3 Activation Triggers Apoptosis in Tumor Cells

The main mechanism through which high TLR3 expression is associated with good prognosis in tumors is apoptosis. Multiple studies have described the ability of TLR3 to induce apoptosis in different in vitro tumor models, mostly through activation of caspases. In breast cancer cell lines, it has been shown that TLR3, once stimulated with its ligand Poly(I:C), leads to Stat1 phosphorylation and strong TRIF-dependent production of IFN-β, together with NF-κB activation and release of pro-apoptotic cytokines. The use of targeted siRNAs showed the specificity of the observed cell death pathways [49]. Similar results were also detected in prostate cancer cell lines. For instance, LNCaP cells treated with Poly(I:C) activate caspase 9 and simultaneously present a biphasic Akt dephosphorylation, which appeared to be responsible for Poly(I:C)-induced antitumor effect [50]. Gambara and colleagues have shown that Poly(I:C) stimulation of the same prostate cancer cell line, LNCaP, leads to IRF3 phosphorylation and an increase in Noxa levels, which promote finally caspase 3 activation and apoptosis, with antitumor response also in vivo [51]. A very recent study showed that TLR3 is also implicated in apoptosis in non-small-cell lung cancer (NSCLC). Histological analyses of biopsies from patients affected by lung adenocarcinoma put into straight correlation the expressions of caspase 3 and TLR3. Such findings were observed also in vitro upon Poly(I:C)/IFN-α stimulation in TLR3 expressing lung tumor cell lines. Along with apoptosis induction in cancer cells, CD103^+^ subpopulation of lung-infiltrating immune cells, but not alveolar macrophages, were activated in an in vitro co-culture system [37].

Although several evidences indicate a TLR3-dependent activation of caspases -3 and -9, one well described apoptotic pathway triggered by TLR3 activation is the extrinsic pathway. Estornes and colleagues demonstrated that Poly(I:C) interaction with TLR3 promotes the binding of TRIF and the subsequent recruitment of RIP1 and caspase 8, to finally trigger apoptosis in lung cancer cell lines [52]. Association of TLR3 with caspase 8 was also observed by Feoktistova et al., who reported the formation of a so-called ripoptosome driven by Poly(I:C)-induced TLR3 activation and caspase-8 recruitment [53]. A further study on prostate cancer models reported that stimulation of TLR3 with Poly(I:C) promoted the activation of the protein kinase C (PKC)-α/JNK-p38 pathway, with subsequent activation of caspase 8 and increase in apoptosis. Interestingly, TLR3-related apoptosis appeared to be positively influenced by AR levels, but not by p53 status [54]. Another protein influencing Poly(I:C)-induced apoptosis was survivin. Indeed, in TLR3-positive head and neck squamous cell carcinoma (HNSCC), the synthetic dsRNA reduced survivin levels in a dose- and TLR3-expression-dependent manner [55].

Interestingly, it has been shown that pro-apoptotic caspase 8 activation downstream TLR3 might depend on the expression of intracellular antiapoptotic molecules, such as a cellular inhibitor of apoptosis (cIAPs) and cellular FLICE-like inhibitory protein (c-FLIP). Indeed, melanoma cells appeared to be insensitive to Poly(I:C)-induced apoptosis but underwent cell death when Poly(I:C) was combined with the inhibitor of protein synthesis cycloheximide, which decreased cIAP1 levels [56]. A similar pattern was also observed in NSCLC and OSC cells, in which TLR3-related apoptosis was induced only upon the combination of Poly(I:C) with paclitaxel, a reported inhibitor of c-FLIP [36].

A further pro-apoptotic mechanism linked to TLR3 activation was described in neuroblastoma. Indeed, it has been demonstrated that TLR3 activation by Poly(I:C) synergizes with 13-cis-retinoic acid in the treatment of high-risk neuroblastoma, through the activation of retinoic acid receptor beta (RARβ). This synergy promoted mitochondrial stress response and increased apoptosis, leading to retarded tumor growth in a mouse xenograft model [57].

Taken together, multiple studies have shown a strong ability of TLR3 to promote caspase-dependent apoptosis in cancer cells, once the receptor is activated, with beneficial outcome in in vitro and in vivo tumor reduction.

### 2.3. TLR3 in Tumor Progression and Metastasis

Despite positive effects on apoptosis, a contrary role of TLR3 in tumor progression and invasiveness has also been proposed. For instance, Jia et al. demonstrated that activation of TLR3 increases cancer stem-cells (CSC) markers and mammosphere-like structure in breast cancer cells cultured in the presence of Poly(I:C). Such result was also confirmed in vivo since treatment with Poly(I:C) decreased tumor size in mice bearing breast tumor, but in parallel enriched for breast CSCs, which appeared strongly tumorigenic when isolated and cultured in vitro. Moreover, this study showed that TLR3 promotes CSC phenotype through the concomitant activation of Wnt/β-catenin and NF-κB signaling pathways [58]. Although the study by Jia and colleagues was well conducted and the phenotype driven by Poly(I:C) stimulation was evident, the authors did not present any experimental evidence that Poly(I:C)-induced effects are exclusively dependent on TLR3 and not on other dsRNA inducible pathways (e.g., MDA5). Therefore, we believe that these observations need further investigation. A different study reported the up-regulation of TLR3 in cancerous intestinal epithelial cells (IEC) with metastatic potential. The addition of Poly(I:C) induced the production of CXCL10 and triggered TLR3 to promote the invasive capability of metastatic IECs [59]. TLR3 was also demonstrated to play a key role in the formation of the pre-metastatic niche in the lungs in vivo. Comparison of TRL3-/- and WT mice showed that TLR3 expression is essential for chemokine production and neutrophils recruitment, features that are critical in the pre-metastatic niche. In fact, lung epithelial TLR3 was activated by exosomal RNAs derived from the primary tumor, stimulating NF-κB, ERK, and p38 pathways to secrete chemokines [38]. In the prostate cancer PC3 cell line, it has been illustrated that TLR3 activation promotes HIF-α expression and nuclear translocation, resulting in increased VEGF synthesis and secretion together with protection from apoptosis [60]. In parallel, TLR3 activation-dependent HIF-α up-regulation appeared to induce changes in cell metabolism and lactate accumulation in prostate cancer cells. Moreover, the authors of this study showed that Poly(I:C) stimulation in benign prostate cells leads to an increase in the use of metabolites, which was described as the typical behavior of cells at the beginning of the neoplastic transformation [61].

Summed up, studies on breast, prostate, lung, and intestinal carcinomas support the role of TLR3 activation in tumor progression via enhanced cancer stemness, metastasis, and metabolic sustainment.

### 2.4. Targeting TLR3 and Therapy Resistance

Given the up-regulation of TLR3 in most cancer types and given the ability of this receptor to trigger apoptosis, together with its capacity to activate the immune system cells, TLR3 might be considered as an important therapeutic target for the treatment of cancer.

TLR3 agonist Poly-ICLC (Hiltonol), which has been previously demonstrated to boost innate immune response in patients [62], was reported to impair the progression of hepatocellular carcinoma when combined with sorafenib, through the activation of NK and CD8^+^ T cells [63]. Further studies have highlighted how the combination of TLR3 agonists with commonly used antitumor therapeutics can improve the outcome. For instance, Ding et al. demonstrated that TLR3-high OSCC cells are more resistant to cisplatin treatment and the pretreatment with Poly(I:C) increases the sensitivity of the tumors to cisplatin-based chemotherapy both in vitro and in vivo. Such a response is driven by the inhibitory effect of Poly(I:C) on ABC transporters translated into decreased drug efflux from cancer cells [44]. Similarly, the combination of cisplatin with Poly(I:C) was also effective in inducing cell death in pleural mesothelioma cell lines [40]. Furthermore, Poly(I:C) was also used in combination with paclitaxel in drug-resistant colon cancer cells, with a successful effect on tumor cytotoxicity through IFN-β secretion [64]. An in vivo study illustrated that Poly(I:C) improves tumor growth retardation driven by radiotherapy in a murine lung cancer cell line (LLC-OVA) with intrinsic resistance to radio- and immunotherapy. Intraperitoneal injection of Poly(I:C) one day before irradiation of LLC-OVA tumor enhanced the tumor shrinkage and, by the use of TLR3- and TICAM-1-deficient mice, the authors demonstrated that the effect was dependent on the activation of TLR3-TICAM-1 pathway [65]. Recently, it has been shown that treatment with Poly(I:C) alone can decrease the tumor weight in mice injected with lung cancer cells [66]. Gierlich et al. have recently evidenced the importance of Poly(I:C) in combination with prostaglandin E2 and TLR7/8 agonist R848, in the maturation of DCs, which have a role in antigen presentation in cancer vaccines. The study showed that these DCs have high migratory ability and priming efficacy, together with increased cytokines production [67].

Interestingly, TLR3 agonist ARNAX has been demonstrated to induce activation and infiltration of tumor-specific cytotoxic T lymphocytes and relieve the resistance to PD-L1 blockade without systemic production of cytokines or IFNs. Indeed, Takeda and colleagues used a cancer vaccine, containing a tumor-associated antigen (TAA) to ARNAX, for the treatment of murine T cell lymphoma by subcutaneous injection, thus inducing CD8^+^ T cell priming through activation of the TLR3-TICAM-1-IRF3-IFN-β pathway in DCs. ARNAX-TAA alone and more in combination with the anti-PD-L1 antibody was able to suppress tumor growth also in cells with innate resistance to immunotherapy. Such effect was due to the change in the population of tumor-infiltrating immune cells, in fact, upon ARNAX administration, Th1-type cells were found in the tumor microenvironment [68].

In a context in which TLR3 is expressed in the endosomal compartment of cells, it is reasonable to question the ability of the agonists to reach their target receptor, especially in the case of systemic administrations. Research has already started to optimize TLR3 ligands internalization inside cells of interest. For example, Schau and colleagues [69] developed a system, which was able to deliver dsRNA TLR3 agonist, Riboxxol, specifically to PSCA-positive tumor cells. This system consisted of neutravidin conjugated to mono-biotinylated dsRNA and human mono-biotinylated anti-PSCA single-chain antibody derivatives. The nanoparticle-like assembly was named Rapid Inducer of Cellular Inflammation and Apoptosis (RICIA) and it was able to successfully enter target cells, which produced type-I IFN response and underwent apoptosis in vitro. In vivo, RICIA nanoparticles did not show adverse effects, but the inhibition of tumor growth was modest, underlining that the full potential of such delivery systems should be further optimized.

Controversial to the reported antitumor role of TLR3, a few recent studies have demonstrated that TLR3 expression and activation may be also associated with resistance to antitumor drugs. The study of Jia et al. not only illustrated an enrichment of CSCs in breast cancer upon TLR3 stimulation, but these CSC-like cells appeared to be more resistant to the common chemotherapeutic drug, such as paclitaxel and doxorubicin [58]. Moreover, Chuang et al. showed in vitro that TLR3-high HNSCC cells have increased resistance to cisplatin compared to TLR3-low cells. Such pattern was confirmed on TLR3 knockout cells, which presented significant cytotoxicity in response to cisplatin treatment. Moreover, TLR3-high cells also appeared more tumorigenic when injected into mice [70]. Although the majority of the previous studies underline the potential of TLR3 as a target in antitumor therapy, it appears clear that this receptor can also sustain carcinogenicity in commonly used anticancer therapeutic strategies. Whether this dual behavior can be related to cancer type or individual characteristics, it is useful to closely monitor the outcome of chemotherapies in patients in terms of TLR3 expression and response to better understand the role of the receptor in each particular case.

## 3. Conclusions

**Scheme 1:**
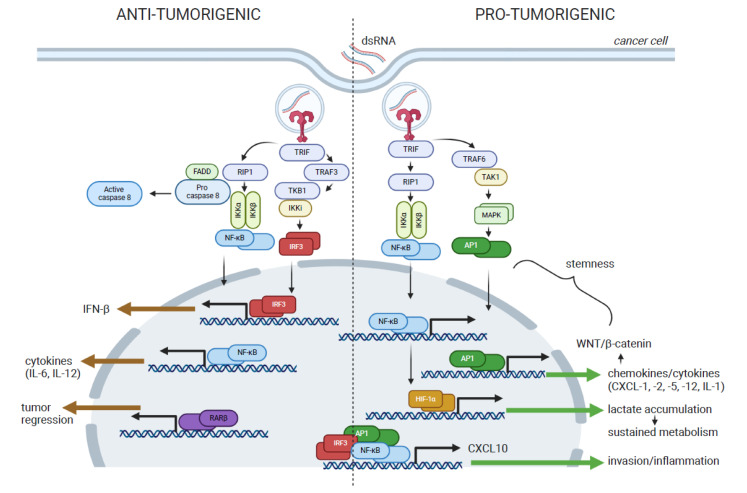
TLR3 has a dual role in tumor fate. Internalization and binding of specific ligand to TLR3 in the endosomes of tumor cells lead to either antitumorigenic or pro-tumorigenic outcomes. Activation of downstream pathways including IRF3, NF-κB, or RARβ has a benefic effect on tumor regression, through the release of cytokines, IFN-β, or active caspases (such as caspase-8). On the other hand, TLR3 activation can stimulate NF-κB or MAPK-dependent effectors which activate pro-survival pathways (such as WNT) and promote chemokine production, with overall positive effect invasion, stemness, and sustainment of tumor growth. Created with Biorender.com.

TLR3 is an endosomal receptor with a well-demonstrated antiviral function in immune and epithelial cells. In recent years, this receptor has also been reported to be expressed in cancer cells and has been proposed as a marker of tumor progression in solid tumors. On the other hand, TLR3 activation can induce apoptosis in tumor cells which has been extensively demonstrated. Furthermore, the ability of the receptor to promote cell death has been widely exploited by researchers, with the attempt to improve pre-existing anticancer therapies, either through the boost of an immune response in the tumor microenvironment or through direct targeting of cancer cells. However, TLR3 appears to have a dual role in tumors, since studies have associated TLR3 levels with either good or poor clinical outcome in different cancer types. Such a controversial “friend and foe” characteristic is linked to the apparent ability of the receptor to promote on one side apoptosis and on another side metastasis and resistance to chemotherapeutic agents (Scheme 1). Overall, we believe that the contradictory effects of the TLR3 pathway activation in tumor tissues could be in part associated with the plasticity of cancer cells. Furthermore, the complexity of the tumor microenvironment, together with the well-known heterogeneity of tumor cells, might also influence the outcome of TLR3 activation. We also strongly consider that the use of proper tools (e.g., validated antibodies and functional experiments) is crucial to obtain more reliable results for clinical application and to offer a better understanding of the mechanism through which TLR3 can determine the clinical fate of anticancer therapies.

## Figures and Tables

**Table 1:** Summary of TLR3 expression and association with clinical outcome in human cancer types. OSC, oral squamous cell; ESC, esophageal squamous cell; Ab, antibody; mAb, monoclonal antibody; pAb, polyclonal antibody; n/a, not available; IHC, immunohistochemistry; WB, western blot; RT-PCR, reverse transcription PCR; RNA-seq, RNA sequencing.

Tumor Type	Sample and Cell Type	TLR3 Expression	Detection Method	Antibody/Primers	TLR3 Localization	Clinical Outcome with High TLR3	Ref.
Prostate cancer	primary tumor	overexpressed	IHC, RT-PCR	commercial mAb clone TLR3.7; primer sequences reported	cytoplasm	poor prognosis	[31]
Breast cancer	primary tumor	overexpressed	IHC, WB, RT- PCR	commercial mAb clone TLR3.7; primer sequences reported	n/a	poor prognosis	[32]
Breast cancer	primary tumor	overexpressed	IHC	in-house mAb clone 40F9.6	cytoplasm	clinical trial; good response to dsRNA in TLR3+ patients	[33]
Breast cancer	TCGA database	poorly expressed	RNA-seq	n/a	n/a	no prognostic value association with ER-/PR-negative breast cancer	[34]
Melanoma	cells isolated from patients	expressed	flow cytometry, WB	commercial mAb clone TLR3.7	n/a	TLR3-dependent apoptosis induction	[35]
Lung cancer	primary tumor	overexpressed	IHC	commercial pAb (PromoCell)	cytoplasm and membrane	n/a; TLR3-dependent apoptosis	[36]
Lung cancer	primary tumor	overexpressed	IHC, microarrays	in-house mAb clone 40F9.6	cytoplasm and nuclei	good prognosis of adenocarcinomaonly	[37]
Lung cancer	primary tumor	overexpressed	IHC	commercial mAb clone 40C1285.6	nuclei	poor prognosis; association with pre-metastatic niche	[38]
Malignant pleural mesothelioma	primary tumor	overexpressed	IHC	commercial pAb (PromoCell)	cytoplasm and membrane	n/a	[39]
Malignant peritoneal mesothelioma	primary tumor	expressed	flow cytometry	commercial mAb clone 1205C5	n/a	n/a; synergy of TLR3 agonist and cisplatin	[40]
Hepatocellular carcinoma	primary tumor	poorly expressed	IHC	commercial mAb clone TLR3.7	cytoplasm and membrane	good prognosis	[41]
Hepatocellular carcinoma	primary tumor	poorly expressed	IHC, WB, RT-PCR	commercial pAb (PromoCell), primer sequences reported	cytoplasm	good prognosis	[30]
Renal cancer	primary tumor and metastasis	overexpressed	IHC, microarray, WB, RT-PCR	commercial mAb clone 40C1285.6	cytoplasm	n/a; growth inhibition by TLR3 ligand	[42]
Ovarian cancer	primary tumor and in vitro cell lines	expressed	IHC, RT-PCR	commercial pAb; primer sequences reported	n/a	n/a; association with serous papillary tumors	[43]
OSC carcinoma	primary tumor, lymph node metastasis	overexpressed	IHC, RT-PCR	Abcam anti-PELI3 E3 ligase Ab used as readout with relation to TLR signaling; discontinued Ab; primer sequences n/a	nuclei	association with higher stage and lymph node metastasis	[44]
OSC carcinoma	primary tumor	overexpressed	IHC	commercial Ab	n/a	poor prognosis	[45]
ESC carcinoma	primary tumor	overexpressed	IHC, RT-PCR	commercial mAb; primer sequences reported	cytoplasm and membrane	association with depth of invasion and lymph node metastasis	[46]
ESC carcinoma	primary tumor	overexpressed	IHC	commercial mAb clone 40C1285.6	cytoplasm and nuclei	no prognostic value	[47]
Gastric cancer	primary tumor	overexpressed	IHC	commercial mAb clone TLR3.7	cytoplasm and membrane	poor prognosis	[48]
